# Influence of polyethylene terephthalate (PET) microplastic on selected active substances in the intramural neurons of the porcine duodenum

**DOI:** 10.1186/s12989-024-00566-w

**Published:** 2024-02-06

**Authors:** Ismena Gałęcka, Natalia Szyryńska, Jarosław Całka

**Affiliations:** 1 Department of Epizootiology, Faculty of Veterinary Medicine, University of Warmia and Mazury in Olsztyn, Oczapowskiego 13, 10-719 Olsztyn, Poland; 2 Department of Histology and Embryology, Faculty of Veterinary Medicine, University of Warmia and Mazury in Olsztyn, Oczapowskiego 13, 10-719 Olsztyn, Poland; 3 Deparment of Clinical Physiology, Faculty of Veterinary Medicine, University of Warmia and Mazury in Olsztyn, Oczapowskiego 13, 10-719 Olsztyn, Poland

**Keywords:** Enteric nervous system (ENS), Neurotransmitter, Gastrointestinal tract, Swine, Plastic, Cocaine and amphetamine regulated transcript, Galanin, Neuronal nitric oxide synthase, Substance P, Vesicular acetylcholine transporter, Vasoactive intestinal peptide

## Abstract

**Background:**

Currently, society and industry generate huge amounts of plastics worldwide. The ubiquity of microplastics is obvious, but its impact on the animal and human organism remains not fully understood. The digestive tract is one of the first barriers between pathogens and xenobiotics and a living organism. Its proper functioning is extremely important in order to maintain homeostasis. The aim of this study was to determine the effect of microplastic on enteric nervous system and histological structure of swine duodenum. The experiment was carried out on 15 sexually immature gilts, approximately 8 weeks old. The animals were randomly divided into 3 study groups (n = 5/group). The control group received empty gelatin capsules once a day for 28 days, the first research group received daily gelatin capsules with polyethylene terephthalate (PET) particles as a mixture of particles of various sizes (maximum particle size 300 µm) at a dose of 0.1 g/animal/day. The second study group received a dose ten times higher—1 g/animal/day.

**Results:**

A dose of 1 g/day/animal causes more changes in the enteric nervous system and in the histological structure of duodenum. Statistically significant differences in the expression of cocaine and amphetamine regulated transcript, galanin, neuronal nitric oxide synthase, substance P, vesicular acetylcholine transporter and vasoactive intestinal peptide between control and high dose group was noted. The histopathological changes were more frequently observed in the pigs receiving higher dose of PET.

**Conclusion:**

Based on this study it may be assumed, that oral intake of microplastic might have potential negative influence on digestive tract, but it is dose-dependent.

## Background

### Microplastic

Currently, society and industry generate huge amounts of plastics worldwide, including polyethylene, polypropylene, polyurethane, polyvinyl chloride, polyethylene terephthalate, polystyrene and polyamide. Each year, humanity increases the production of plastics, as exemplified by the value of the plastics market, whose value for 2022 was USD 609.01 billion, and the predicted value for 2030 will be approximately USD 811.57 billion [1]. Plastics are used massively in every aspect of life, from food storage and clothing production to children’s toys. Plastics are closely related to the continuous generation of waste by-products and production residues. The above characteristics and applications are closely related to the presence of microplastics. Microplastic present in the environment can be divided, depending on its origin, into primary (produced in such a form) and secondary (formed as a result of an impact on plastic). Hartmann et al. proposed a plastic debris classification system based on (I) chemical composition, (II) physical state, (III) solubility, (IV) size, (V) shape and structure, (VI) color and (VII) origin [2]. According to the aforementioned classification, plastic particles from 1 to 1000 µm should be considered microplastics [2]. The European Food Safety Authority (EFSA) report defines particle sizes between 0.1 and 5000 µm [3] and Frias et al. between 1 and 5000 µm [4]. The information provided indicates the need to standardize the terminology, to avoid misunderstandings or misleading public opinion and the consumers. The massive use of plastics in practically every sphere of human life contributes to the release of microplastics into water [5–7], soil [8] and air [5, 9]. The COVID-19 pandemic and the large-scale use of personal protective equipment (such as masks and gloves) resulted in an increase in the number of microparticles in the environment [10, 11]. The plastic for food and beverages packaging promotes their entry into the digestive tract [12–14]. Plastic waste, regardless of its characteristics, has become a problem that civilization has to face. For example, the European Union, as part of the European Green Deal, plans to reduce the landfilling of plastic waste to 55% by 2030. Despite the undertaken initiatives, the ubiquity of microplastics in the environment is obvious, but their influence on animals and humans is still not fully understood [15–17]. The scientific problem mentioned earlier depends on several factors, such as the type (polystyrene, polyethylene terephthalate, polyethylene, polyvinyl chloride, etc.), particle size (macroplastic, microplastic or nanoplastic), route of entry into the body (aerogenic, transdermal, oral), exposure time, research model or dose [17–19].

### The enteric nervous system

The digestive tract is one of the first barriers between pathogens, xenobiotics and living organisms. Its proper functioning is extremely important in order to maintain homeostasis, but to make it possible, it was necessary to create mechanisms regulating the work of the digestive tract at the neuronal and endocrine levels [20]. Lymphatic tissue associated with the gastrointestinal tract (GALT) or the enteric nervous system (ENS) is such a mechanisms. The enteric nervous system, which is part of the autonomic nervous system, regulates its work by controlling the muscles, blood vessels or the secretory activity of the mucosal epithelium [21]. It consists of ganglia and nerve fibers lying in the wall of the esophagus, stomach, small and large intestines, pancreas, bile ducts and gallbladder [22]. The change in neurotransmitter coding is influenced by a number of factors, including age [23, 24], intestinal microbiota [25, 26], pathological conditions such as diabetes [27, 28] or inflammatory bowel disease (IBD) [29–31], substances which we take more or less consciously, such as non-steroidal anti-inflammatory drugs [32, 33] and bisphenols [34]. In pigs, which are a model organism in human biomedical research and whose anatomical structure resembles the human anatomical structure in the intestines [35–38] we can distinguish three plexuses: myenteric (MP) and submucous, which is further subdivided into the outer (OSP) and inner (ISP) submucous plexus [39]. By secreting neuronally active substances in these plexuses, the ENS can stimulate or inhibit the work of the digestive tract in response to the current conditions of the microenvironment. The myenteric plexus is mainly responsible for the motility of the digestive tract and the submucous plexus for secretory activity [40]. From the neurotransmitters found in the ENS, six were selected to study their activity in response to microplastics. Each of them performs an important function in the ENS to maintain homeostasis [41, 42]. Cocaine and amphetamine-regulated transcript (CART) regulates intestinal motility (inhibits stomach emptying) and inhibits the secretion of gastric juice, but the exact role in the digestive tract is unknown [43]. Galanin (GAL) is responsible for inhibiting intestinal motility or emptying the stomach, participates in inflammatory processes (exhibiting neuroprotective effects, mediates and participates in interactions between the immune and neurological systems and has antiproliferative and proapoptotic effects) and regulates secretory activity (inhibits the secretion of somatostatin, insulin, glucose and stomach acid) [44–46]. Nitric oxide (NO), an atypical neurotransmitter, is generated by nitric oxide synthase (NOS). There are three types of nitric oxide synthases—neuronal (nNOS), inducible (iNOS), and endothelial (eNOS). Nitric oxide, which is produced by these enzymes, is responsible for inhibiting intestinal motility and promoting vasodilation [47] and is also involved in inflammatory processes [48]. Neuropeptide substance P (SP), as a representative of tachykinins induced the secretion of water and electrolytes. It has a pro-inflammatory effect by stimulating the production of cytokines such as IL-1β, IL-6, IL-8, and TNF-α [49–51]. The vesicular acetylcholine transporter (VAChT) is considered the main marker of cholinergic neurons [52]. The function of acetylcholine is secretory stimulation and the regulation of blood flow [40, 53]. It also exhibits anti-inflammatory activity [54, 55]. The role of vasoactive intestinal peptide (VIP) in the gastrointestinal tract is to dilate blood vessels, increase intestinal blood flow and stimulate intestinal secretion [56, 57]. Like substance P, it has an immunomodulatory effect [50, 58].

### Implications in food chain and research needs

The identification of microplastic particles in samples of animal feed [59], food [60–62] and feces [63–68] is clear evidence of the ongoing exposure of the gastrointestinal tract. From the point of view of initiatives such as Farm to Fork (F2F), the presence of microplastics in the food chain threatens the health and safety of consumers. Even despite the actions taken regarding the sustainability of food systems or the European Green Deal, eliminating the threat related to microplastics seems to be unrealistic at present. Studies on porcine cell cultures have been reported in the available literature reports [69, 70]. However, due to the multitude of biochemical processes and the metabolic complexity of the digestive system, it is necessary to conduct in vivo studies. To date, no such studies have been undertaken and the available reports describe only the occurrence of particles in the lungs of pigs from the natural environment [71]. It should be emphasized that the domestic pig (*Sus scrofa domestica*) is considered one of the better models in human biomedical research, especially of the gastrointestinal tract [35, 37, 38]. The doses used in the studies presented were intended to be overload doses and do not reflect the actual amounts of molecules typically ingested by humans and animals. Although these doses were of a cognitive nature, they may prove to be adequate in the future. The use of such high doses allowed for the definition of polyethylene terephthalate microplastics (PET-MP) and their effects on the neuroplasticity of the enteric nervous system in the duodenum to be studied.

## Results

### Myenteric plexus

**Fig. 1:**
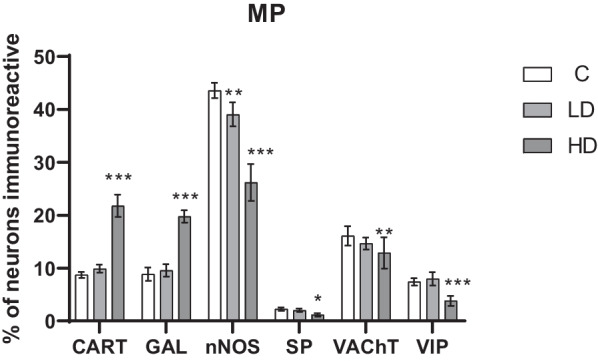
Percentage of neurons immunoreactive in the myenteric plexus. Percentage (*mean* ± *SEM*) of neurons immunoreactive for CART, GAL, nNOS, SP, VAChT and VIP in control (C) and in animals receiving low (LD) and high dose (HD) of MP-PET. *p < 0.05, **p < 0.01, ***p < 0.001 indicate statistically significant differences in the expression of the tested substances in relation to the control group

**Fig. 2:**
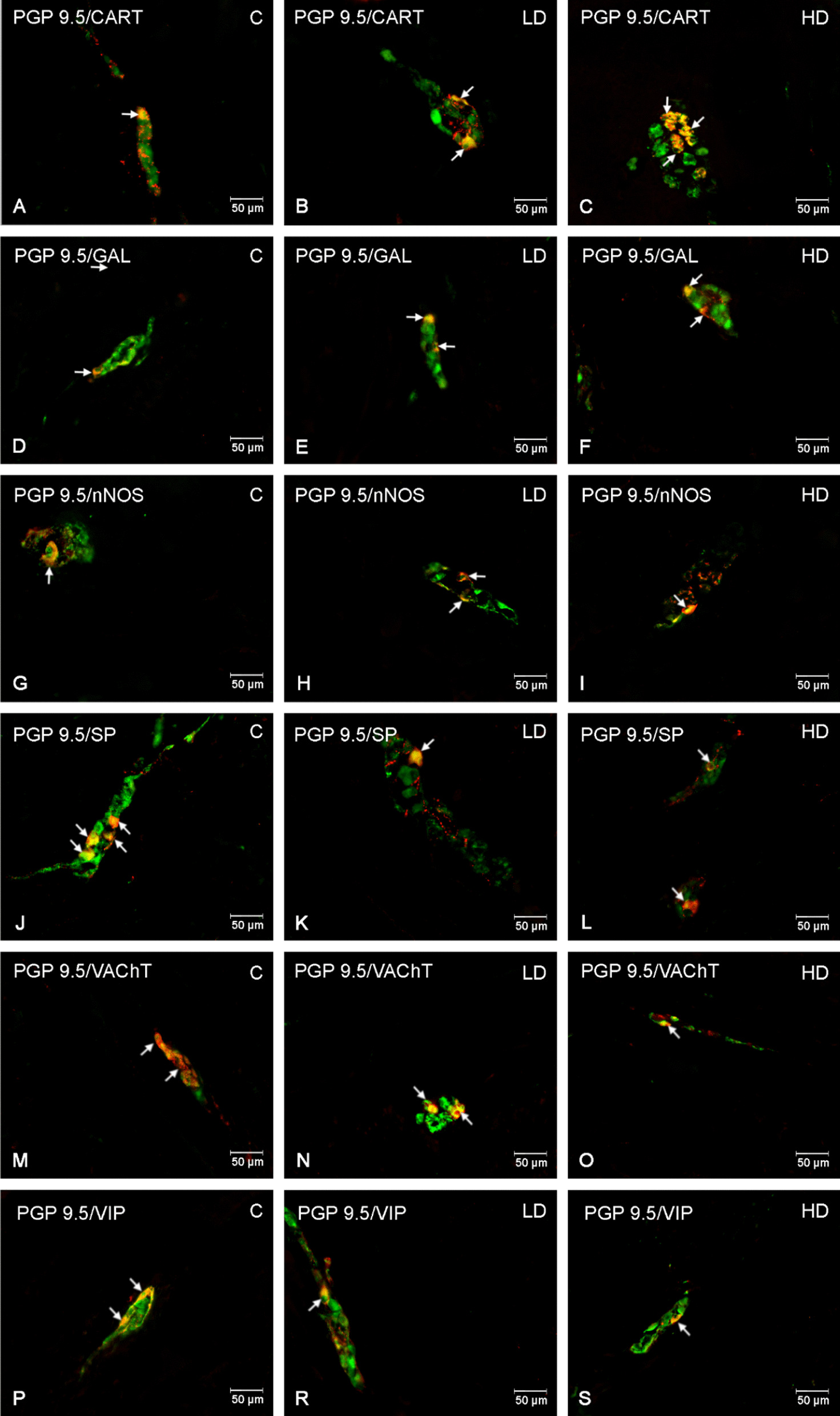
Myenteric plexus of porcine duodenum. Fluorescence microscope image showing the distribution of neurons immunoreactive to PGP 9.5—used as a pan-neuronal marker and CART, GAL, nNOS, SP, VAChT, VIP in the control group (C) (**A**, **D**, **G**, **J**, **M**, **P**), after administration of low (LD) (**B**, **E**, **H**, **K**, **N**, **R**) and high (HD) (**C**, **F**, **I**, **L**, **O**, **S**) doses of MP-PET. All photographs were made by overlapping green and red fluorescent channels (green for PGP 9.5 and red for CART, GAL, nNOS, SP, VAChT and VIP, respectively). Neurons immuno-positive to a particular substance studied are indicated with arrows

In the myenteric plexus (MP) the low dose significantly reduced only the number of nNOS positive neurons (*p* < 0.01), which was also the most numerous population in the C group. In the remaining cases, the low dose did not change the size of the population (*p* > 0.05). In the case of CART and GAL, there was a significant increase in the population of positive neurons in the HD group compared to the C group (*p* < 0.001). The high dose has the weakest impact on the size of population of VAChT and SP-positive neurons, where the significance was (*p* < 0.01) and (*p* < 0.05), the low dose did not cause statistically significant differences (*p* > 0.05). In neurons positive for nNOS and VIP, a decrease in neuronal population was observed (*p* < 0.001). The exact results are presented in Figs. 1, 2.

### Outer submucous plexus

**Fig. 3:**
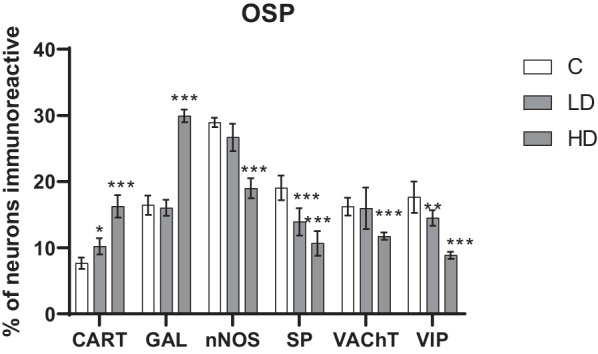
Percentage of neurons immunoreactive in the outer submucous plexus. Percentage (*mean* ± *SEM*) of neurons immunoreactive for CART, GAL, nNOS, SP, VAChT and VIP in the control (C) and in animals receiving low (LD) and high dose (HD) of MP-PET. *p < 0.05, **p < 0.01, ***p < 0.001 indicate statistically significant differences in the expression of the tested substances in relation to the control group

**Fig. 4:**
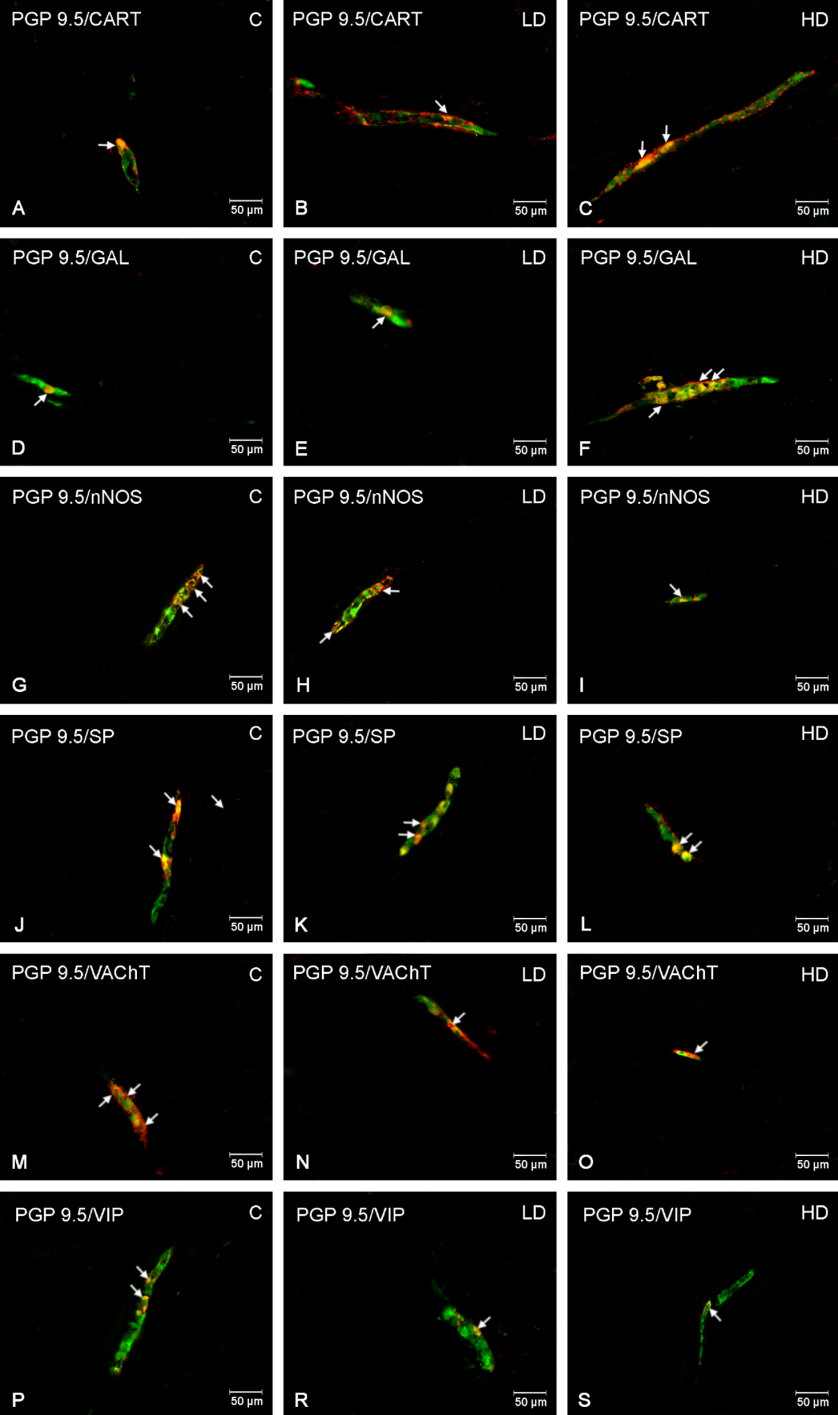
Outer submucous plexus of the porcine duodenum. Fluorescence microscope image showing the distribution of neurons immunoreactive to PGP 9.5 —used as a pan-neuronal marker and CART, GAL, nNOS, SP, VAChT, VIP in the control group (C) (**A**, **D**, **G**, **J**, **M**, **P**), after administration of low (LD) (**B**, **E**, **H**, **K**, **N**, **R**) and high (HD) (**C**, **F**, **I**, **L**, **O**, **S**) doses of MP-PET. All photographs were made by overlapping green and red fluorescent channels (green for PGP 9.5 and red for CART, GAL, nNOS, SP, VAChT, and VIP, respectively). Neurons immuno-positive to a particular substance studied are indicated with arrows

There were several similarities with the MP found in the outer submucous plexus (OSP). Altough nNOS-positive cells also in this plexus constituted the largest population in the control group, microplastic supplementation resulted in a decrease in their activity only in the HD group (*p* < 0.001). The least numerous population were CART-positive neurons. In animals receiving low (*p* < 0.05) and high (*p* < 0.001) doses of microparticles, an increase in the number of CART-positive neurons was observed. The high dose increased the percentage of GAL-positive neurons (*p* < 0.001), with no differences due to the low dose (*p* > 0.05). In the case of SP and VIP, a significant decrease in neuronal activity was observed, regardless of the dose taken. The activity of VAChT was significantly reduced (*p* < 0.001) after high dose supplementation. The exact results are shown in Figs. 3, 4.

### Inner submucous plexus

**Fig. 5:**
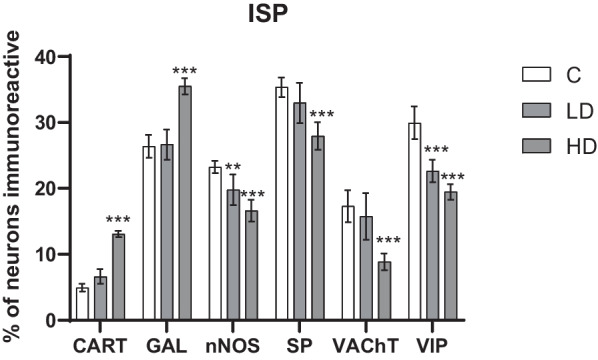
Percentage of neurons immunoreactive in the inner submucous. Percentage (*mean* ± *SEM*) of neurons immunoreactive for CART, GAL, nNOS, SP, VAChT and VIP in the control (C) and in animals receiving low (LD) and high dose (HD) of MP-PET. *p < 0.05, **p < 0.01, ***p < 0.001 indicate statistically significant differences in the expression of the tested substances in relation to the control group

**Fig. 6:**
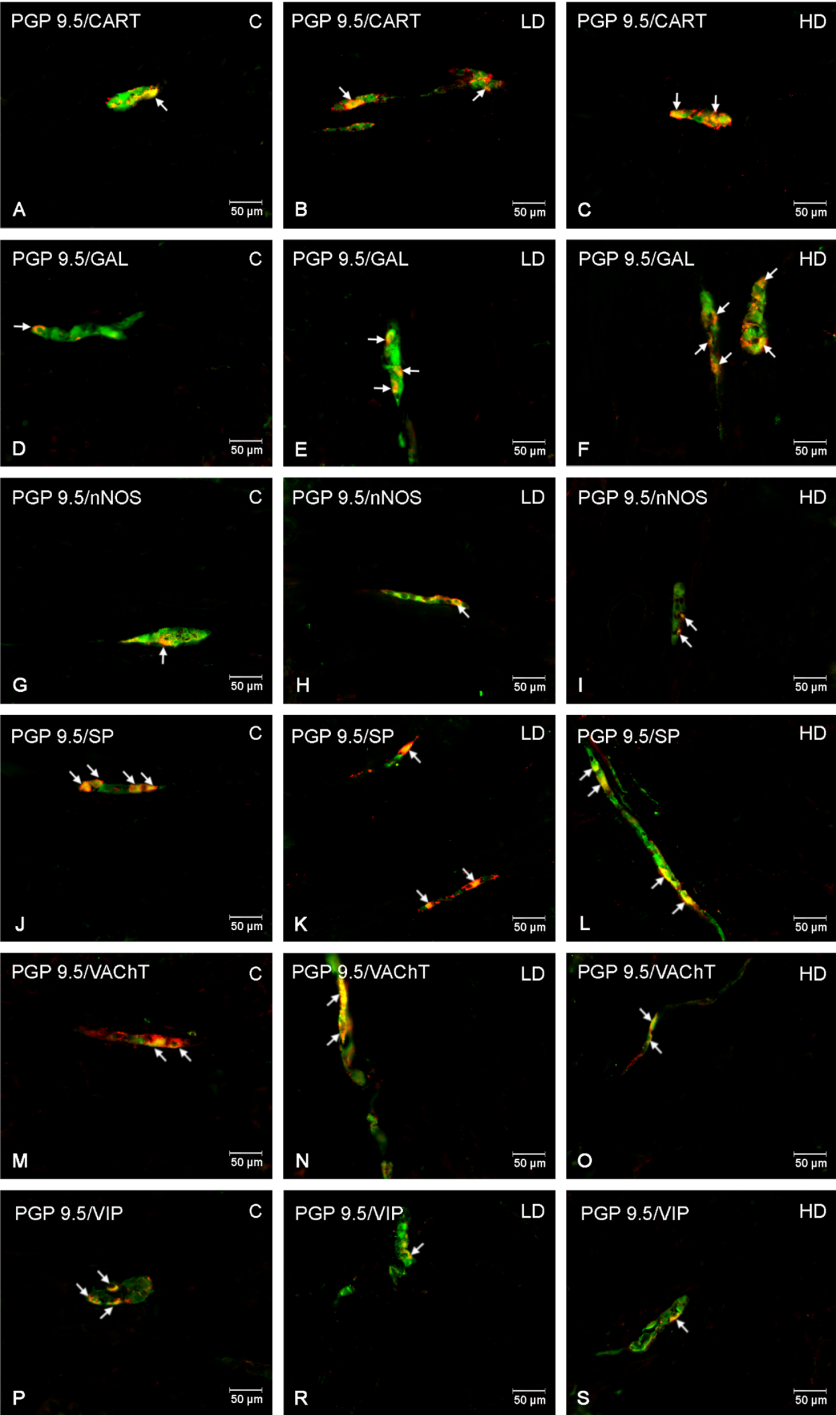
Inner submucous plexus of porcine duodenum. Fluorescence microscope image showing the distribution of neurons immunoreactive to PGP 9.5 —used as a pan-neuronal marker and CART, GAL, nNOS, SP, VAChT, VIP in the control group (C) (**A**, **D**, **G**, **J**, **M**, **P**) after administration of low (LD) (**B**, **E**, **H**, **K**, **N**, **R**) and high (HD) (**C**, **F**, **I**, **L**, **O**, **S**) doses of MP-PET. All photographs were made by overlapping green and red fluorescent channels (green for PGP 9.5 and red for CART, GAL, nNOS, SP, VAChT, VIP respectively). Neurons immuno-positive to a particular substance studied are indicated with arrows

In the inner submucous plexus, a high dose of MP-PET caused a decrease in SP (*p* < 0.001), nNOS (*p* < 0.001), VAChT (*p* < 0.001) and VIP (*p* < 0.001) positive neurons and an increase in CART (*p* < 0.001) and GAL (*p* < 0.001) positive neurons. The low dose had an effect on the decrease only in the case of nNOS (*p* < 0.01) or VIP (*p* < 0.001) positive neurons, without affecting the number of CART, GAL, SP and VAChT positive neurons (*p* > 0.05). The exact results are shown in Figs. 5, 6. Detailed results of one-way ANOVA for all convolutions are presented in Table 1.

**Table 1:** Detailed results of one-way analysis of variance

	For the model				For research groups			
	*df*	*MS*	*f*	*p* Value *	*df*	*MS*	*f*	*p* Value *
MP								
CART	1	145.03	1300.21	< 0.001	2	13.55	121.48	< 0.001
GAL		155.47	1489.21	< 0.001		12.07	115.62	< 0.001
nNOS		1016.88	4503.16	< 0.001		20.75	91.89	< 0.001
SP		2.80	154.76	< 0.001		0.09	5.02	< 0.01
VAChT		163.56	1318.69	< 0.001		0.65	5.25	< 0.01
VIP		31.50	530.01	< 0.001		1.30	21.85	< 0.001
OSP								
CART	1	98.09	983.90	< 0.001	2	4.90	49.19	< 0.001
GAL		342.78	2140.04	< 0.001		16.16	100.88	< 0.001
nNOS		478.51	2584.45	< 0.001		7.01	37.87	< 0.001
SP		164.71	1328.36	< 0.001		4.55	36.71	< 0.001
VAChT		164.42	1324.79	< 0.001		1.68	13.53	< 0.001
VIP		142.01	1215.64	< 0.001		5.00	42.77	< 0.001
ISP								
CART	1	50.75	685.37	< 0.001	2	4.61	62.30	< 0.001
GAL		710.16	3454.10	< 0.001		7.26	35.34	< 0.001
nNOS		308.74	1951.41	< 0.001		2.97	18.79	< 0.001
SP		803.64	3710.00	< 0.001		3.80	17.55	< 0.001
VAChT		148.91	1249.38	< 0.001		5.14	43.11	< 0.001
VIP		439.24	2429.47	< 0.001		7.26	40.15	< 0.001

MP—myenteric plexus, OSP—outer submucous plexus, ISP—inner submucous plexus, CART—cocaine and amphetamine regulated transcript, GAL—galanin, nNOS—neuronal nitric oxide synthase, SP—substance P, VAChT—vesicular acetylcholine transporter, VIP—vasoactive intestinal peptide, *df*—degrees of freedom, *MS*—mean squares, *f*—Fisher F ratio, * statistical significance at *p* < 0.05.

### Histological analysis

**Fig. 7:**
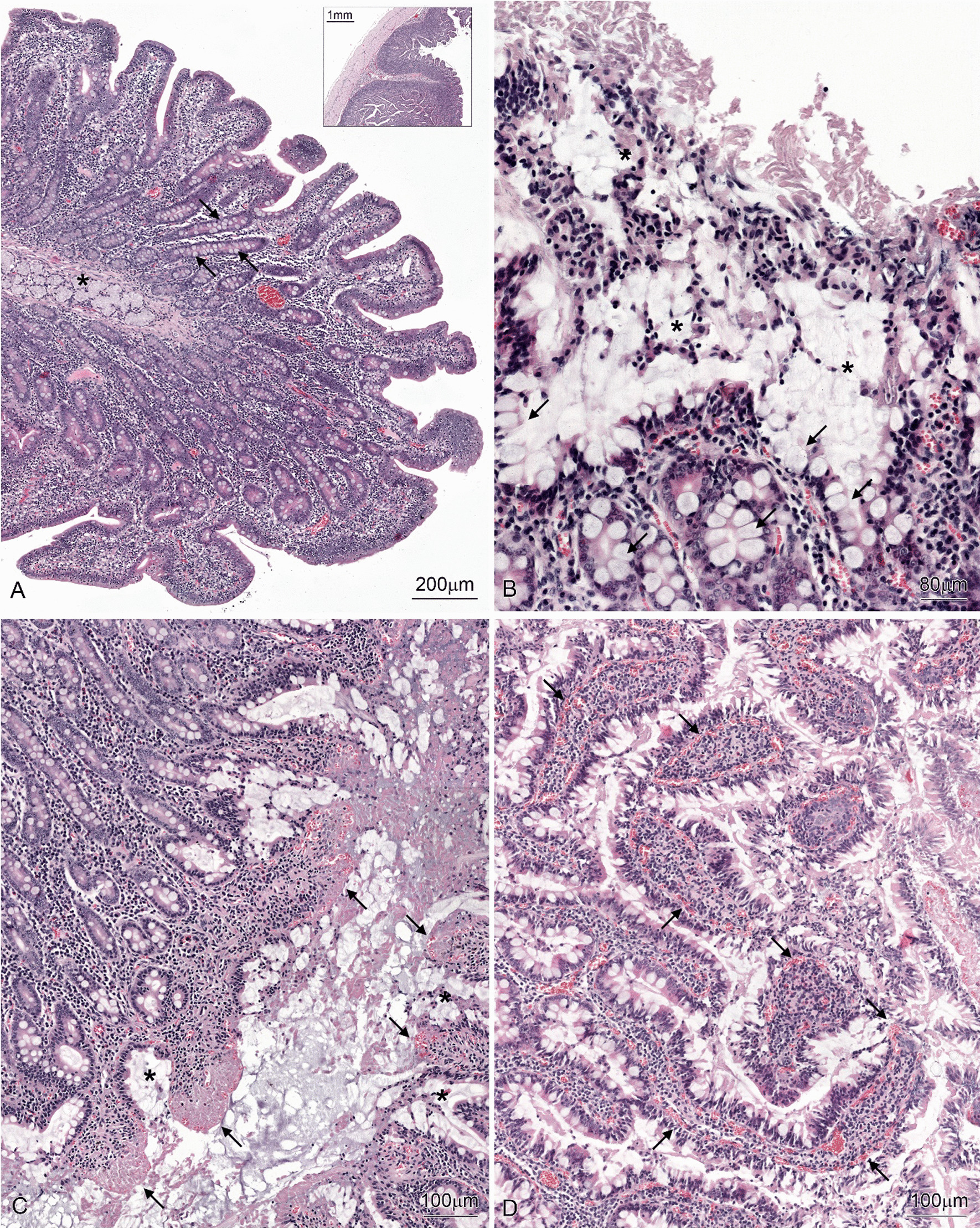
Histological structure of the duodenum in a C, LD and HD group. **A** Note short and irregular villi, numerous goblet cells (arrows) in the crypts, and the Brunner’s glands (asterisk). The insert shows the section though the entire wall of the duodenum. **B** Note presence of numerous goblet cells (arrows) in the epithelium and the thick mucus layer containing cellular debris (asterisks). **C** Note the detachment of enterocytes from the villi (arrows) and the presence of mucus with cellular debris between the villi (asterisks). **D** Note numerous blood-containing vessels (arrows)

The structure of the duodenum in the C group was typical for this segment of the pig intestine. The mucosa formed short and irregular villi. Goblet cells were frequently noted in the mucosal epithelium, especially in the crypts. The lamina propria contained numerous lymphocytes and plasma cells. The submucosa was filled by the well-developed Brunner’s glands. Pathological changes were not found in the investigated sections of the duodenum of control pigs. In contrast, focal changes with the desquamation of enterocytes and the accumulation of large amounts of mucus were frequently observed in the duodenum sections from the LD and HD groups. The loss of enterocytes occurred both on the apical and lateral surfaces of the villi. The characteristic feature of the villi lacking the epithelium was the presence of blood-containing vessels in their tips. The mucus with cellular debris covered the mucosa forming a thick layer on its surface and frequently filled the spaces between villi. In the affected parts of the intestine, enormous accumulations of goblet cells were noted in the epithelium of the upper parts of crypts and the basal parts of the villi. The lamina propria showed hyperaemia. The pathological changes were more frequently observed in the pigs receiving a high dose of MP-PET (Fig. 7).

A morphometric analysis showed differences in the length of villi, which were significantly lower in the LD and HD groups compared to the control groups and in the pigs receiving a high dose than in those receiving a low dose of MP-PET. The depth of the crypts did not differ between the groups. The thickness of the mucosa was significantly lower in pigs receiving a high dose of MP-PET than in the C group. There were no differences in the thickness of the submucosa and muscularis externa (Table 2).

**Table 2:** Statistical analysis of histological study

Parametr (µm)	C	LD	HD
Length of villi	356.38 ± 46.57	315.81*** ± 45.14	272.14*** ± 45.22
Deep of crypts	594.25 ± 85.62	585.87 ± 68.27	561.37 ± 98.34
Thickness of mucosa	915.97 ± 115.79	920.00 ± 113.66	779.90*** ± 114.14
Thickness of submucosa	232.58 ± 44.99	219.94 ± 44.77	221.64 ± 49.79
Thickness of muscularis externa	423.05 ± 54.44	411.20 ± 63.64	430.5*** ± 56.14

The length of villi, the deep of crypts, the thickness of mucosa, the thickness of submucosa, and the thickness of the muscularis externa in the duodenum of pigs from the C, LD and HD group (*mean* ± *SD*). ***p < 0.001 indicate statistically significant differences in the expression of the tested substances in relation to the control group.

### Ultrastructural study

**Fig. 8:**
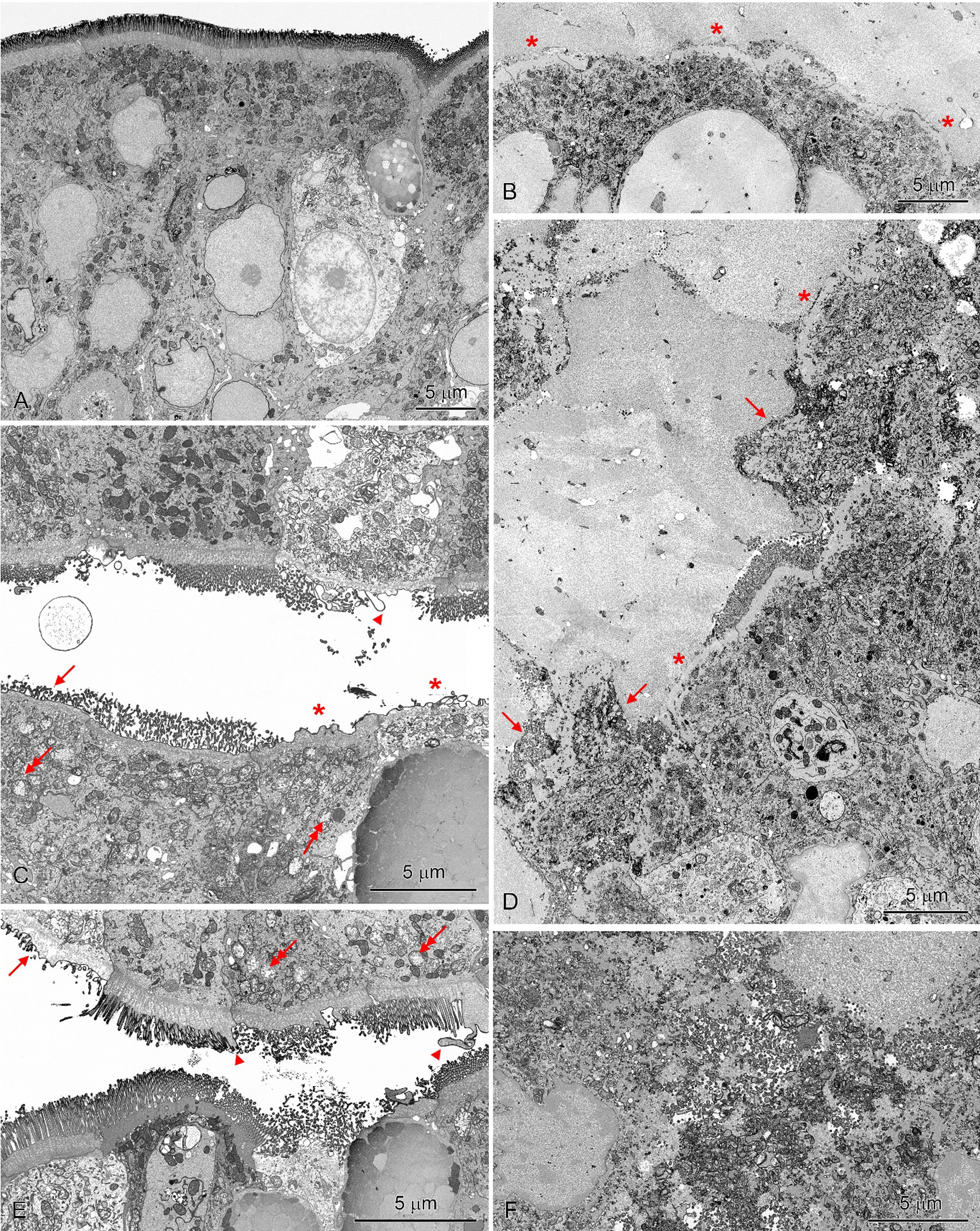
Ultrastructure of the duodenum epithelium in a C group (A), LD (B, C) and HD group (D, E, F). **A** Note numerous, long, regularly arranged microvilli on the apical surface and numerous mitochondria with a moderate electron density of the matrix in the upper part of absorptive cells. **C**, **E** Note shortage of microvilli (arrows), loss of microvilli (asterisks), protrusions on the apical surface of endothelial cells (arrow heads) and swollen mitochondria (double arrows). **B**, **D**. Epithelium covered by a thick layer of mucus. A lack of microvilli (asterisks) and damage of epithelial cells (arrows). F. Damaged cells in the area covered by a thick mucus layer

In group C, the intestinal villi were covered by a continuous epithelial cell layer comprising mainly absorptive cells and goblet cells (Fig. 8A). The apical surface of absorptive cells formed numerous, long, regularly arranged microvilli. The prominent bundles of microfilaments descend from the microvilli into the terminal web. The upper part of the cytoplasm contained numerous mitochondria with moderate electron density of matrix and well-developed cristae. The goblet cells were less frequent than absorptive cells and had a typical structure with an accumulation of large, irregular granules in their widened upper part.

The intestinal villi showed pathological changes in LD (Fig. 8B, C) and HD (Fig. 8D–F) pigs. The extent and character of these changes differed between samples and were correlated with the presence of an enormous, thick layer of mucus on the mucosa surface. In the areas without accumulation of mucous, adsorptive cells showed shortage of microvilli and partial or complete loss of microvilli, although the epithelium still retained its integrity and continuity (Fig. 8C, E). The cells formed variable protrusions (in shape and size) on the apical surface (Fig. 8C, E). As a consequence, the brush border lacked its regular organization. The changes in microvilli correlated with the reduction of the terminal web and changes in the cytoplasm of the absorptive cells. The cells with damaged microvilli frequently comprised mitochondria with an electron-lucent matrix, sometimes obviously swollen (Fig. 8C, E). Some absorptive cells showed vacuolization.

**Fig. 9:**
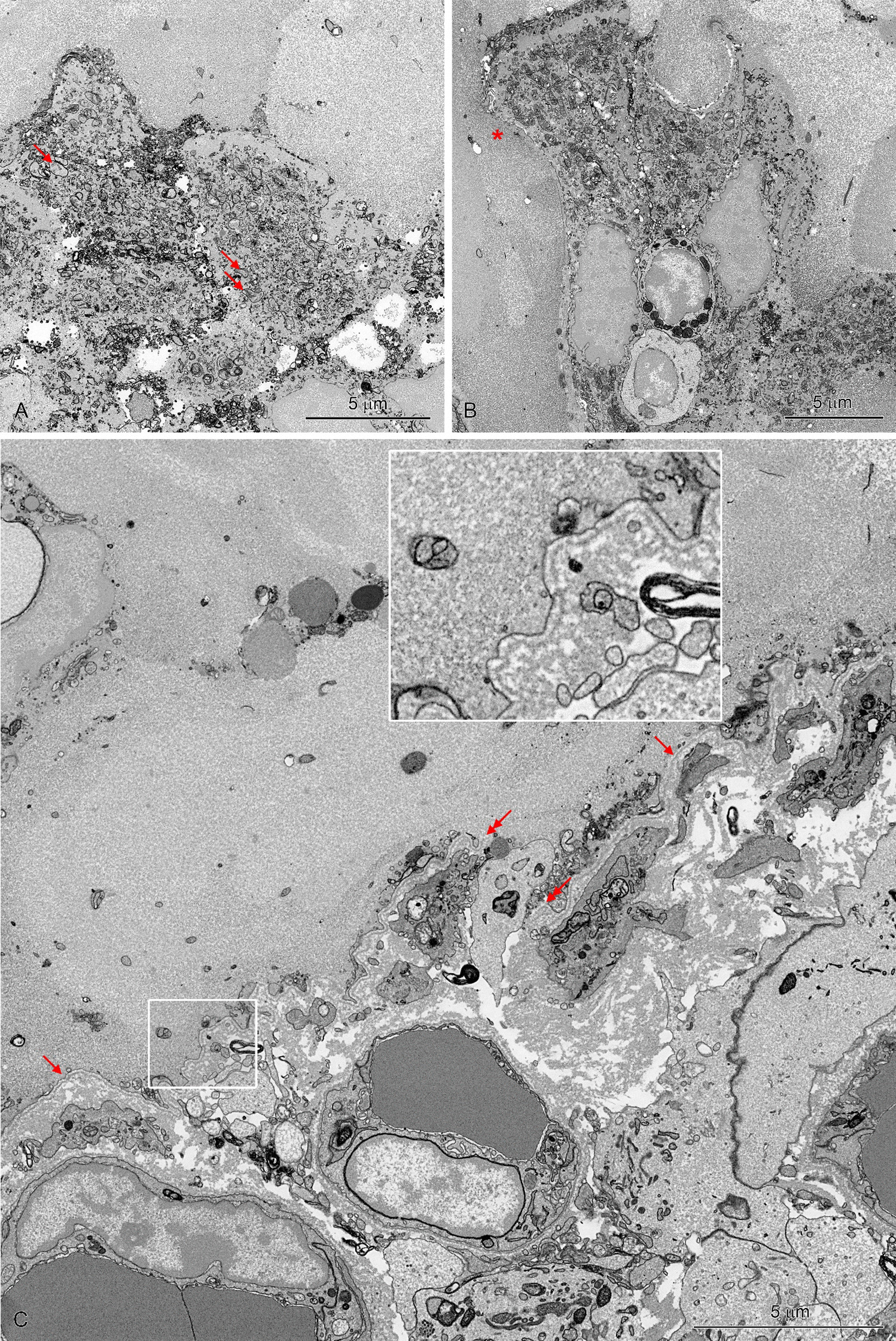
Ultrastructure of the duodenum epithelium covered a thick mucus layer in the HD group. **A** Note swollen mitochondria (arrows) and numerous vesicles in the cytoplasm of damaged cells. **B** A lack of epithelium continuity (asterisk). **C** Area with a complete loss of epithelial cells. Note that the lamina propria is separated from the mucous by the basement membrane (arrows, insert—a part of the image at higher magnification). Double arrows show a break in the basement membrane

Much more serious damage to the intestine epithelium was observed in the areas of mucosa covered by a thick mucus layer. The severity of the injury enabled three degrees of damage to be distinguished. The first degree included almost a complete loss of microvilli and damage to the apical parts of a few adsorptive cells, but with the remained continuity of the epithelium (Fig. 8B). Numerous goblet cells occurred in these areas. The second degree of injury was defined by the presence of many damaged cells with swollen mitochondria and numerous vesicles in the cytoplasm (Figs. 8D, F, 9A, B). Spillage of degraded cell remnants into the lumen resulted in breaks in the epithelial continuity. The layer of mucus was extremely thick and contained cellular debris. The third degree included the areas with a complete loss of epithelial cells (Fig. 9C). The connective tissue of the lamina propria was separated from the mucous by the basement membrane, which remained intact in most of the damaged area. The foci without the basement membrane were sporadically found.

**Fig. 10:**
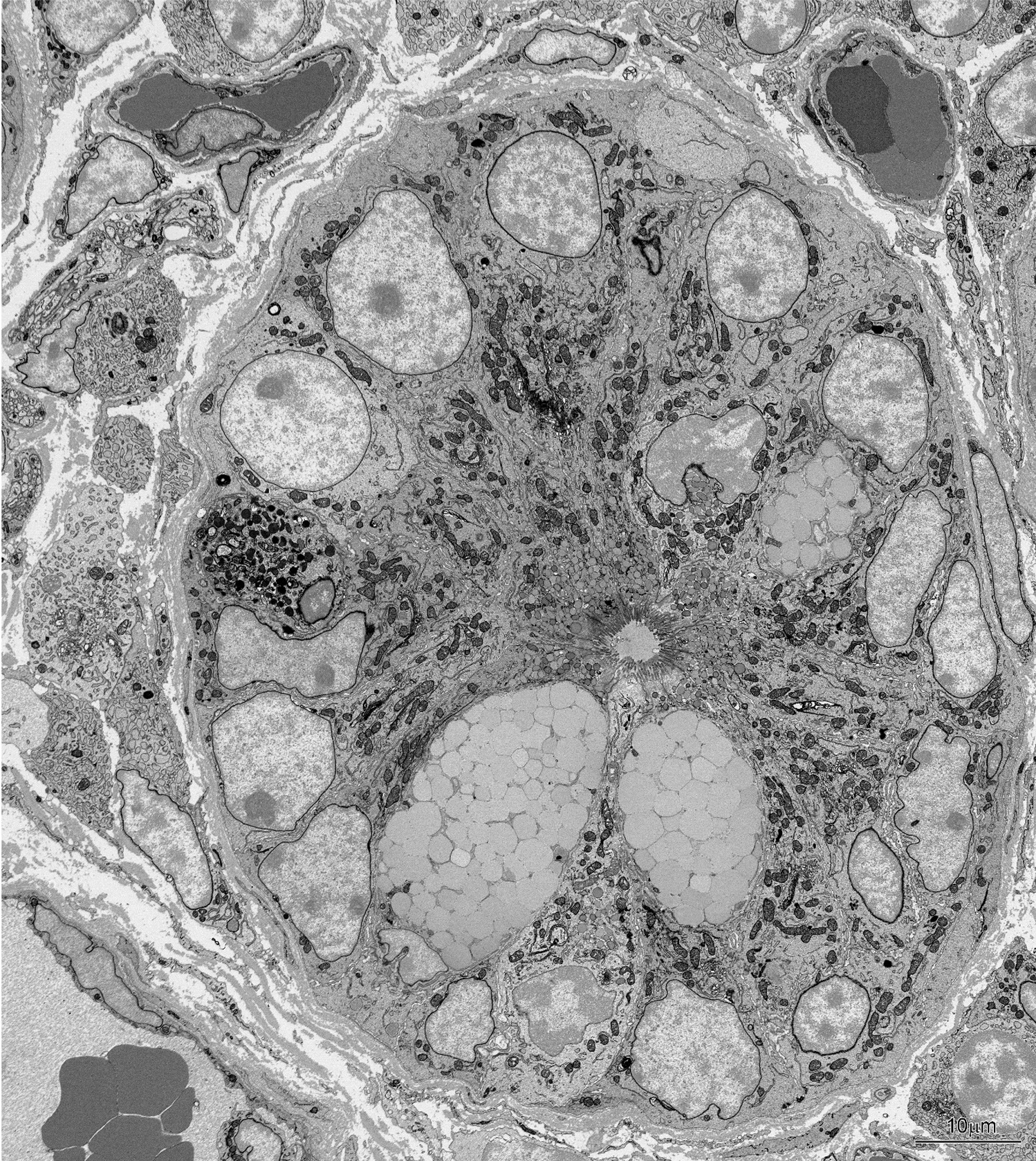
Ultrastructure of intestinal glands located beneath the area with damaged villi in the HD group. Note the normal organization of cells

The ultrastructure of intestinal glands located beneath the areas with damaged villi, showed no pathological alternations (Fig. 10).

## Discussion

Based on the available literature, it can be concluded that the presented research shows for the first time the effect of a cumulative dose of microplastic on the enteric nervous system in a domestic pig. To date, studies have been conducted mainly on mice, rats or aquatic organisms [17, 72–74]. Based on the available literature, the generalized conclusion can be drawn that the effect of microplastics on animals is influenced by several factors. It has been reported that microplastics in vertebrates can accumulate in internal organs such as the ovaries, testes [72, 75], intestines, kidneys, liver [76–79] and lungs [71, 76]. In humans, microplastics have been identified in the liver [80], intestines [81] lungs [82], placenta [83, 84] milk [85] and sputum [86]. Studies conducted by Donkers et al. [87] on advanced in vitro models representing the lungs and intestines using various types of micro- and nano-plastics, aimed at demonstrating the potential of these molecules to induce cytotoxic and pro-inflammatory effects. They confirmed the ability to translocate plastic particles through the lung and intestinal epithelium, as well as the ability to activate pro-inflammatory cells [87]. The ability to penetrate the intestinal barrier suggests that orally ingested microplastics may spread throughout the body through the bloodstream and accumulate in tissues. This phenomenon can be described as plasticemia—the presence of microplastic particles in the peripheral blood. However, the accumulation of microparticles can lead to organ dysfunction and impairment of one system or even the whole organism.

Senathirajah et al. predicted that the standard consumer is exposed to 0.1–5 g/week of microplastic (approximately equivalent to the weight of a credit card) [88]. Pletz claims that these data are overestimates [89]. Cox et al. [14] evaluate the annual consumption at the level of 39,000 to 52,000 particles depending on age and gender. Mohamed et al. conducted a probabilistic assessment trying to estimate how many particles will be ingested by a standard consumer over a lifetime and reported 553 particles/capita/day for children and 883 particles/capita/day for adults) [90]. A large discrepancy can be observed in the mentioned research. This may result from only hypothetical assumptions, burdened with error and high variance and even socioeconomic status. This state of knowledge confirms that it is impossible to clearly determine what effect microplastics have on living organisms, because it depends on too many variables, such as the shape, size or number of particles, and without knowing the amount of ingested particles it is impossible to determine how it affects the body and, most importantly, how to effectively counteract these effects. In addition, as it is not a biological substance, standard analytical methodology is difficult to apply. Larger plastic fragments can cause physical tissue damage, such as the lateral line of fish [91], and smaller ones can penetrate deep into the body, e.g. to the intestines [92]. On the other hand, there are reports that microplastics do not have a significant effect on fish development as was supposed [93, 94]. Undoubtedly, microplastics and the correlated environmental burden are part of a One Health issue [95, 96], and thus requires a multidisciplinary approach to manage the risks posed to humans, animals and the environment.

Previous studies have shown that the enteric nervous system, due to its plasticity, adapts to the influence of adverse factors such as chemicals or inflammation of the intestines and diabetes [32, 33, 97–100]. This is possible due to a dense network of neurons in the intestines, which form plexuses and depending on the conditions, modify the secretion of neuronally active substances. The proper functioning of the enteric nervous system affects not only the digestive system, but also the entire body. In the case of neurodegenerative diseases such as Parkinson's disease, dysfunction in the functioning of the ENS has been observed, and is suspected as the potential origin of this disease [101]. The results presented in this article suggest that the occurrence of microplastics may be correlated with inflammation in the intestine [102]. The confirmation of the above may be a positive correlation between the concentration of microplastics in the feces and the severity of IBD symptoms [102]. Currently, however, it is not possible to determine whether the presence of IBD increases the retention of microplastics in the intestines or whether microplastics increase inflammatory symptoms [102].

The presented results confirm the adaptation of the enteric nervous system in response to PET-MP doses expressed by changes in the population of neurons CART, GAL, nNOS, SP, VAChT and VIP positive. The severity of changes varied depending on the examined plexus. In the duodenum, food passed from the stomach is mixed with bile from the gallbladder and pancreatic juice. This allows the neutralization of stomach acid and the absorption of nutrients, vitamins and minerals. Changes in chemical coding in this section of the digestive tract may affect the absorption of substances in further sections of the intestine. The digestive process initiated in the stomach changes the surface of plastics, generating fine particles on their surface [103]. A molecule that passes through the digestive tract may show different morphological features (changes in size, shape and chemical properties). This may result in a different effect on the ENS depending on the examined part of the gastrointestinal tract. In order to confirm this thesis, it is suggested to perform studies determining the population of positive neurons in various sections of the digestive tract, as well as to determine the exact changes that microplastic undergoes during the in vivo digestion process.

Cocaine and amphetamine-regulated transcript have a gastric acid-blocking effect [43]. It has neuroprotective properties [104]. Under the influence of bisphenol A, a decrease in the population of CART-positive neurons was observed in the duodenum [34], unlike indomethacin [33], acrylamide [105] or glyphosate [99]. The significant increase in CART neurons in the present study can be explained by the neuroprotective effect. Inhibiting the secretion of gastric acid may reduce the exposure of microplastics to digestive enzymes, and therefore less likely to cause changes in the ingested molecules and altered interaction in further sections of the digestive tract.

Vasoactive intestinal peptide induces relaxation of smooth muscles and stimulates the secretion of water, electrolytes and secretion of bicarbonates by the pancreas. It is responsible, like CART, for the inhibition of gastric acid secretion. Through these actions it increases intestinal motility [56, 106]. Vasoactive intestinal peptide has a neuroprotective effect through glial cells that produce cytokines. The decrease in VIP-positive neurons noted in the present study was also observed with exposure to bisphenol A [34] and lipopolysaccharides [107]. However, most inflammatory conditions result in an increase in VIP activity due to its immunomodulatory and neuroprotective properties [108]. In the present studies, PET-MP reduced the activity of VIP in the duodenum, which may have a neurodegenerative effect on the neurons of the examined segment of the intestine.

Galanin exhibits neuroprotective properties and acts as an inhibitory neuromodulator [46]. An increase in GAL activity has been noted in pigs after long-term supplementation with a non-steroidal anti-inflammatory drug [33] acrylamide [109] or in dogs with IBD [31]. Galanin may affect the motility of the gastrointestinal tract by inhibiting acetylcholine and SP via excitatory GAL-R_1_ receptors [110]. In the present results, the population of GAL-positive neurons increases and the population of VAChT and SP-positive neurons decreases. This confirms the assumptions of the inhibitory and anti-inflammatory effects of GAL in the ENS. Reduced intestinal motility may result in the retention of intestinal contents containing microplastics, which may promote the entry into the bloodstream of molecules able to overcome the intestinal barrier.

The vesicular acetylcholine transporter (VAChT) is considered the main marker of cholinergic neurons [52]. The function of acetylcholine is secretory stimulation and the regulation of blood flow [40, 53]. It also exhibits anti-inflammatory activity by lowering the level of pro-inflammatory cytokines [54, 55]. A decrease in the population of VAChT-positive neurons is observed in the case of exposure to glyphosate [99] or bisphenol A [34], while an increase is observed after supplementation with acrylamide [109] or in mice with Huntington's disease [111]. Bisphenol A is one of the components that is used in the production of plastics, and it causes a similar effect as the tested PET-MP. A reduced level of acetylcholine may promote the development of inflammation in the intestines.

The activity of SP is mainly correlated with the occurrence of inflammation [112] and is associated with pain symptoms [113]. It stimulates the production of pro-inflammatory cytokines through the neurokinin-1 receptor (NK_1_R) [51], and regulates the transport of ions and fluids through the epithelium to the intestinal lumen [114]. A decrease in SP activity was noted with long-term exposure of pigs to naproxen [32] and in rats after administration of ulcerogenic agents [115], with multiple organ dysfunction syndrome [116] and diabetes [117, 118]. This has been associated with the relaxation of the intestinal muscle leading to motility disorders or with the formation of ulcers in the duodenum. In humans with constipation [119] or with diabetes, a decrease in SP levels has been observed in the rectal [120] and gastric [121] mucosa. An increase in SP activity can be observed much more often under the influence of various factors causing inflammation in the intestines [33, 34, 109]. The decrease in the number of positive SP in the results presented in the article may result from the degree of inflammation, degeneration or necrosis of neurons under the influence of a toxic agent [106].

The variability in the number of nNOS-positive neurons is an occurrence often observed in gastrointestinal disorders. Nitric oxide is considered one of the main inhibitory factors, causing the relaxation of the intestinal muscles, the inhibition of the secretion of intestinal hormones and the regulation of the transport of water and electrolytes. Because nNOS inhibit the secretion of gastric juice, it has a protective function [122]. Diabetes in pigs [28] and exposure to acetylsalicylic acid [123] causes a decrease in the number of nNOS-positive neurons, while an increase in the population of nitrergic neurons is observed in dogs with IBD [100] and diabetic rats [124]. In diseases where the intestinal mucosa is only slightly damaged, elevated levels of NO are reported [122]. Polyethylene induces NO production in human cell lines with pro-inflammatory properties [125]. Whether NO has pro- or anti-inflammatory properties is not fully understood. The observed decrease in the level of nitric oxide under the influence of PET-MP may adversely affect intestinal neurons.

A decrease in nNOS, VAChT and VIP-positive neurons may be one of the defense mechanisms against microplastics. These neurotransmitters can cause vasodilation, which in the case of inhibition of intestinal motility caused by (among others) the activity of galanin, creates conditions conducive to the absorption of microparticles from the intestines into the bloodstream. Primarily, acetylcholine and SP-secreting neurons are excitatory neurons. They stimulate smooth muscles, increase the secretion of intestinal juice, dilate blood vessels and secrete intestinal hormones [110]. PET-MP in the current study reduced the population of the previously mentioned neurons. This may result in a negative effect on intestinal peristalsis (affecting the MP) and digestive functions (affecting the OSP and ISP). Reducing the activity of SP and VIP may weaken the immune response by inhibiting the activity of macrophages [107]. The presented results correspond with the observation obtained by Szymanska et al. [34] on bisphenol A. Similar population behaviors were noted in the population of VIP and VAChT positive neurons, but different in the SP and GAL populations. Bisphenols are not used in the production of PET packaging, although, other agents used in the production of PET products may act in a similar way. It should be noted that each plastic differs in chemical composition, which may cause convergent or divergent effects on the body. It is worth noting that exposure to microplastics outside of controlled laboratory conditions varies greatly. The mixture of molecules taken orally is not uniform in composition or size. This fact may generate various effects on the enteric nervous system which are different from the results obtained in the current study.

**Fig. 11:**
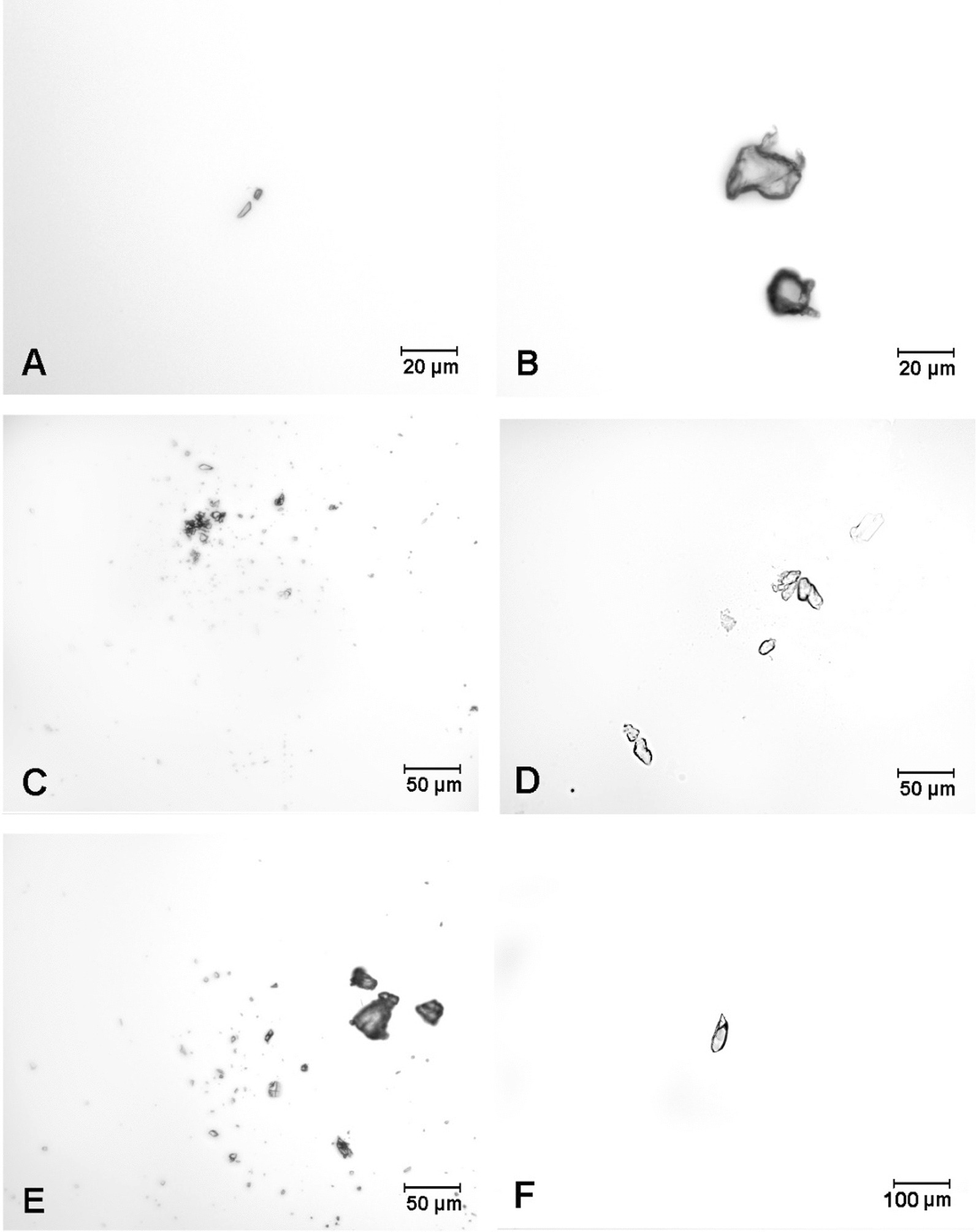
Particles of polyethylene terephthalate used in the experiment

The analysis of histological changes suggests a negative effect caused by the mechanical impact of MP-PET particles on the duodenal mucosa. The diverse shape of MP-PET, including the presence of particles with potentially sharp edges (Fig. 11), increases the likelihood of cell damage, including focal desquamation of enterocytes. Mucus present in the intestines has a protective function and prevents the adhesion of microorganisms [126]. Mice exposed to oral polypropylene showed a decrease in mucus production and the destruction of the intestinal mucosal barrier in response to oxidative damage and altered inflammatory cytokine levels [127]. It can be assumed that MP-PET initially caused the loss of microvilli, to which the body responded with increased mucus secretion as a defense mechanism. In the duodenum of the mouse [128] similarly to the presented study, the proliferation of small blood vessels and infiltration of inflammatory cells such as lymphocytes and plasma cells in the lamina propria were found. The influence of microplastics on the cells of the digestive tract in *Daphnia magna* [129] and *Artemia parthenogenetica* [130] corresponds to the results obtained, i.e. the shortening of the length of the microvilli and their damage. In smaller animals such as *Dapnia* and *Artemia* this may result in stunted growth or increased mortality. In pigs, these effects have not been observed, probably due to a significantly larger area of the gastrointestinal tract and the selected dose was too low to cause such an effect.

## Conclusion

Based on this study, it may be assumed, that oral intake of microplastic might have a potential negative influence on the digestive tract. The changes observed in the chemical coding of the enteric nervous system in the duodenum, along with the structural changes, confirm that the body is taking action to counteract the inflammation caused by the microplastic. Changes are more pronounced in the group of animals receiving microplastics at a dose of 1 g/day than in the group receiving 0.1 g/day. Despite not having precise knowledge about the amount of microplastics taken in by humans, the state that may be dealt with in connection with the widespread use of plastics is illustrated by the current study.

## Material and methods

The aim of this study was to evaluate, how two different doses of polyethylene terephthalate PET (the material most often chosen for packaging beverages and water), affect the enteric nervous system and the morphology of swine duodenum.

### Animals and experimental procedures

The experiment was carried out on 15 sexually immature Pietrain X Duroc breed gilts, approximately eight weeks old, weighing approximately 20 kg, from a farm in Lubawa (Poland). The start of the study was preceded by obtaining consent from the Local Ethics Committee (decision no. 10/2020 of 26 February 2020). All procedures were carried out in accordance with Polish law, which defines the conditions and methods of conducting experiments on animals and the European Community Directive on the ethical use of experimental animals. The animals were kept under standard laboratory conditions (20–22°C, 55–60% relative humidity, 12 h/12 h light-night cycle) in the animal rooms of the Faculty of Veterinary Medicine of the University of Warmia and Mazury in Olsztyn, Poland. The animals were fed twice a day with commercial feed, with unlimited access to water. All plastic items have been removed from the animals environment. The animals were randomly divided into three study groups (n = 5/group). The control group (C) received empty gelatin capsules once a day for 28 days. The first research groups (LD and HD) received daily gelatin capsules with PET particles (cat. no. ES306031/1, Good Fellow) (Fig. 11) as a mixture of particles of various sizes (spherical, fibrous, irregular). For particle analysis, 500 randomly selected particles were subjected to microscopic analysis with the use of Zeiss Axio Imager.M2 fluorescence microscope (Zeiss, Oberkochen, Germany) and software ZEISS ZEN Microscopy Software (Zeiss, Oberkochen, Germany). Randomization was performed by obtaining a representative sample of the entire material from different depth levels and then splitting it into 25 microscope slides. Then, 20 randomly selected particles were measured on each slide. The average length of the larger side was 153.09 µm, *Min* – 1.25 µm, *Max* – 299.75 µm, *SD* – 85.13 and *SEM *– 3.81. The first study group (LD) received at a dose of 0.1 g/animal/day and the second study group (HD) received a dose ten times higher—1 g/animal/day. The low and high dose of microplastics were developed on the basis of the logarithm of the decimal (for log(0,1) =  − 1; for log(1) = 0). The capsules were administered one hour before the morning feeding. The animals were euthanized after a period of 28 days. Premedication was performed using Atropine (0.05 mg/kg *i.m*., Polfa, Warsaw, Poland), followed by induction with xylazine (3 mg/kg *i.m*., Vet-Agro, Lublin, Poland) and ketamine (6 mg/kg *i.m.,* Vetoquinol Biowet, Gorzów Wlkp., Poland). After approximately 20 min, an overdose of sodium pentobarbital (0.6 ml/kg, *iv*., Biowet, Puławy, Poland) was administered. After confirming that life functions had stopped (lack of pupillary reflex, pulse and respirations), the material was immediately collected for further examination. About 4 cm of duodenal fragments (approx. 10 cm away from the pyloric sphincter muscle) were collected for immunofluorescence and histopathological examination.

### Double immunofluorescent staining

To fix the preparations, duodenal fragments were placed in a 4% paraformaldehyde solution (pH 7.4) for one hour, then rinsed with 0.1 M phosphate buffer (pH 7.4, every 24 h, 3 times) and finally transferred to 18% sucrose solution (pH 7.4) for two weeks at 4 °C. The sucrose solution was replaced every 10 days. The duodenal fragments were frozen using a Tissue-Tek O.C. T. (Sakura Finerek USA, Inc., Torrance, CA, USA) and a cryostat (CM 1860, Leica, Germany), cut perpendicularly to the intestinal lumen into 14 µm fragments and affixed to chrome-alum-coated microscope slides. Tissues prepared using this method were kept at -20 °C until staining was performed. After drying the sections at room temperature for 45 min and washing them three times in 0.1 M phosphate-buffered saline (PBS, pH 7.4 for 15 min) samples were blocked with the blocking mixture consisting of 10% horse serum and 0.1% bovine serum albumin in 0.1 M PBS, 1% Triton X-100, 0.05% Thimerosal, and 0.01% sodium azide in room conditions for an hour. The next step involved washing three times with PBS solution. Duodenal sections were covered overnight with a mix of primary antisera PGP 9.5 (panneuronal marker) and CART, GAL, nNOS, SP, VAChT or VIP, at room temperature in a water bath. After 24 h, after three washes with PBS, the sections were incubated for 1 h with Alexa Fluor 488 and Alexa Fluor 546 secondary antibodies. At the end of the procedure, the sections were secured with carbonate-buffered glycerol (pH 8.6) and coverslipped. The list of antibodies used in the procedure along with the concentrations used is provided in the Table 3. Stained sections were analyzed under a Zeiss Axio Imager.M2 fluorescence microscope (Zeiss, Oberkochen, Germany) equipped with the appropriate set of filters. A camera connected to a PC with ZEISS ZEN Microscopy Software (Zeiss, Oberkochen, Germany) was used for image acquisition. To determine the percentage of neurons immunoreactive to selected neuroactive substances, at least 500 PGP 9.5 positive neurons (with a clearly visible nucleus) were counted in each duodenal plexus, which were counted as 100% of the population. Then the filter was changed to visualize CART, GAL, nNOS, SP, VAChT or VIP positive neurons and the percentage population of the selected ones was determined. To avoid double counting of the same neurons where a mixture of the same antibodies was used, the distance between the sections was at least 200 µm.

**Table 3:** Antibodies used in the immunofluorescence method

Antigen	Species	Dilution	Code	Supplier
PGP 9.5	Mouse	1:1000	480012	ThermoFisher Scientific, Waltham, MA, USA
CART	Rabbit	1:16,000	H-003-61	Phoenix Pharmaceuticals, Inc., Burlingame, CA, USA
Gal	Guinea pig	1:2000	T-5036	Peninsula, San Carlos, CA, USA
nNOS	Rabbit	1:3000	PA1-033	ThermoFisher Scientific, Waltham, MA, USA
SP	Rat	1:150	8450-0505	BioRad, Hercules, CA, USA
VAChT	Rabbit	1:2000	H-V006	Phoenix Pharmaceuticals, Inc., Burlingame, CA, USA
VIP	Rabbit	1:2000	ab22736	Abcam, Cambridge, United Kingdom
AF 488	Donkey anti-mouse IgG (H + L)	1:1000	A21202	ThermoFisher Scientific, Waltham, MA, USA
AF 546	Donkey anti-rabbit IgG (H + L)	1:1000	A10040	ThermoFisher Scientific, Waltham, MA, USA
AF 546	Goat anti- guinea pig IgG (H + L)	1:1000	A11074	ThermoFisher Scientific, Waltham, MA, USA
AF 546	Goat anti-rat IgG (H + L)	1:1000	A11081	ThermoFisher Scientific, Waltham, MA, USA

PGP 9.5—(panneuronal marker) protein gene product 9.5, CART—cocaine and amphetamine regulated transcript, Gal—galanin, nNOS—neuronal nitric oxide synthase, SP—substance P, VAChT—vesicular acetylcholine transporter, VIP—vasoactive intestinal peptide, AF—Alexa Fluor.

### Histological study

The tissue was fixed in 4% paraformaldehyde solution in 0.1 M phosphate buffer (pH 7.4) for 48 h. Next, it was dehydrated anethanol (50%, 70%, 90%, 96%, 96%, 99.9%), ethanol 99.9%-xylene mixture (1:1), xylene (3 times) and paraffin (2 times) series (TP 1020, Leica, Germany). The whole process took 23 h. After that, the samples were embedded in paraffin (EG1150, Leica, Germany). The 4 µm-thick sections were prepared using microtome (HM 340E, Microm, Spain) and stained with hematoxylin and eosin method (HE) using a multi-stainer (ST5020 + CV5030, Leica, Germany). The slides were digitalized in Pannoramic 250 Flash scanner (3DHistech, Budapest, Hungary). Morphometric analyses were performed manually using SlideViewer 2.6 software (3DHistech, Budapest, Hungary) and included the length of villi, the crypt deep, the thickness of mucosa, the thickness of submucosa, and the thickness of the muscularis externa. The measurements were performed on three sections per animal, separated from each other by at least 1 cm, in 10 replicates per slide, and the mean values were subjected to a statistical analysis.

### Ultrastructural study

The duodenum samples were immersion-fixed in a mixture of 1% paraformaldehyde and 2.5% glutaraldehyde in 0.2 M cacodylate buffer (pH 7.4) for 2 h at 4 °C. Next, they were washed in the buffer and post-fixed in a solution containing 2% aqueous osmium tetroxide and 1.5% potassium ferrocyanide in 0.15 M cacodylate buffer with 2 mM calcium chloride for 1 h, washed in water and placed in freshly prepared, filtered 1% thiocarbohydrazide solution for 20 min. The samples were then rinsed in water, incubated in 2% osmium tetroxide for 30 min, rinsed again in water and placed in 1% aqueous uranyl acetate for overnight incubation at 4°. The following day, the samples were stained according to Walton’s lead aspartate method for 30 min, washed in water, dehydrated and embedded in Epon 812. The ultrathin sections were cut using PT3D PowerTome ultramicrotome with ASH2 (Boeckeler Instruments, Inc., Tucson, AR, USA) and collected on silicon wafers. The sections were imaged in hierarchical mode using a Sense BSD backscatter electrons detector in SEM Gemini 450 controlled by Atlas 5 software (Carl Zeiss, Oberkochen Germany).

### Statistical analysis

The assumption of linearity and normality was checked before statistical analysis. To study linearity, two-dimensional scatter plots of the analyzed variables were generated. The assumption of normality was validated using histograms and residual normality plots. The following values were calculated for the obtained results: mean (*M*), standard deviation (*SD*) and standard error of the mean *(SEM*). A one-way analysis of variance (ANOVA) was used to show statistically significant differences between the number of neurons immunoreactive to CART, GAL, nNOS, SP, VAChT, VIP, length of villi, deep of crypts, the thickness of mucosa, submucosa and muscularis externa (dependent variables) in individual research groups (qualitative variable). The homogeneity of variance was determined using Levene's test before ANOVA. Post-hoc analysis (Scheffe test) was used to assess statistically significant differences between the individual study groups. Differences were considered statistically significant at *p* < 0.05. Statistical analysis of the obtained results was carried out using the Statistica 13.3 program (TIBCO Software Inc., Palo Alto, USA).

## Data Availability

The datasets used and/or analysed during the current study are available from the corresponding author on reasonable request.

## Abbreviations

AF: Alexa Fluor
C: Control group
CART: Cocaine and amphetamine regulated transcript
*df*: Degrees of freedom
eNOS: Endothelial nitric oxide synthase
ENS: Enteric nervous system
*f*: Fisher F ratio
GAL: Galanin
GALT: Lymphatic tissue associated with the gastrointestinal tract
HD: High dose group
IBD: Inflammatory bowel disease
IL: Interleukins
iNOS: Inducible nitric oxide synthase
ISP: Inner submucous plexus
LD: Low dose group
MP: Myenteric plexus
*MS*: Mean squares
nNOS: Neuronal nitric oxide synthase
OSP: Outer submucous plexus
PBS: Phosphate-buffered saline
PET: Polyethylene terephthalate
PET-MP: Polyethylene terephthalate microplastics
PGP 9.5: Protein gene product 9.5
SP: Substance P
TNF: Tumor necrosis factor
VAChT: Vesicular acetylcholine transporter
VIP: Vasoactive intestinal peptide
